# Combining Photodynamic Therapy and Targeted Drug Delivery Systems: Enhancing Mitochondrial Toxicity for Improved Cancer Outcomes

**DOI:** 10.3390/ijms251910796

**Published:** 2024-10-08

**Authors:** J. P. Jose Merlin, Anine Crous, Heidi Abrahamse

**Affiliations:** Laser Research Centre, Faculty of Health Sciences, University of Johannesburg, Doornfontein, P.O. Box 17011, Johannesburg 2028, South Africa; acrous@uj.ac.za (A.C.); habrahamse@uj.ac.za (H.A.)

**Keywords:** mitochondria, toxicity, cancer, photodynamic therapy, photosensitizer

## Abstract

Cancer treatment continues to be a substantial problem due to tumor complexities and persistence, demanding novel therapeutic techniques. This review investigates the synergistic potential of combining photodynamic therapy (PDT) and tailored medication delivery technologies to increase mitochondrial toxicity and improve cancer outcomes. PDT induces selective cellular damage and death by activating photosensitizers (PS) with certain wavelengths of light. However, PDT’s efficacy can be hampered by issues such as poor light penetration and a lack of selectivity. To overcome these challenges, targeted drug delivery systems have emerged as a promising technique for precisely delivering therapeutic medicines to tumor cells while avoiding off-target effects. We investigate how these technologies can improve mitochondrial targeting and damage, which is critical for causing cancer cell death. The combination method seeks to capitalize on the advantages of both modalities: selective PDT activation and specific targeted drug delivery. We review current preclinical and clinical evidence supporting the efficacy of this combination therapy, focusing on case studies and experimental models. This review also addresses issues such as safety, distribution efficiency, resistance mechanisms, and costs. The prospects of further research include advances in photodynamic agents and medication delivery technology, with a focus on personalized treatment. In conclusion, combining PDT with targeted drug delivery systems provides a promising frontier in cancer therapy, with the ability to overcome current treatment limits and open the way for more effective, personalized cancer treatments.

## 1. Introduction

The Pan American Health Organization (PAHO) states that there will be over 10 million cancer deaths and 20 million new cases of cancer in 2023 [1,2]. Cancer is an intricate illness that includes many modifications in the transcriptome; proteome; and genotypic, genetic, and metabolism categories [3]. Carcinogenic chemicals, biological processes involving thermal radiation, reactive oxygen species (ROS), and an unclear combination of polymerases and phosphodiesterases are among the environmental and lifestyle factors that affect the development of cancer [4]. Due in large part to inadequate chemotherapies and delayed diagnoses, cancer is among the most typical reasons for dying [5,6]. Common cancer treatments such as chemotherapy and targeted therapy are effective but are constrained by recurrent drug resistance and tumor return [7]. In general, there are two mechanisms that lead to cancer medication resistance: intrinsic and acquired factors [8]. Prior to the administration of any medication, mechanisms mediating resistance are responsible for intrinsic resistance, whereas responsive reactions that support tumor cells in their survival after medication therapy in adverse environments are responsible for acquired drug resistance [9]. These adaptive responses include decreased drug absorption or increased drug efflux, insufficient stimulation of apoptosis, and compensatory activation of signal transduction that promotes survival [10]. The development of anti-cancer medications has heavily relied on molecularly targeted treatments, which seem to be primarily ligand-receptor-based associations or enzymes that inhibit a specific target [11].

Recent advancements in cancer biology have shed light on pancreatic ductal adenocarcinoma, including numerous signaling pathways, resistance to therapy that develops gradually, genetic instability, tumor microenvironment, and cellular heterogeneity [12]. Numerous scientific advancements emphasize the critical need for new approaches for cancer treatment in addition to the drug sensitivity strategy of conventional chemotherapy, which only targets a few biochemical pathways [11]. An innovative method for eliminating, without approaching chemoresistance in cancer, is to target a subcellular organelle as opposed to a certain peptide or enzyme, or to neutralize a crucial protein in organelles [13]. To create chemoresistance in cancer treatment, targeting mitochondria to destroy cancer cells might be preferable over ligand-receptor interactions, as the release of cytochrome c (cyt c) from the mitochondria is only a prominent event within the intrinsic induction and apoptosis signaling cascade [14]. Using a combination of light irradiation and photosensitizers (PS), photodynamic therapy (PDT) is a sophisticated approach for tumor ablation at the clinical stage. However, there is a chance that the body will become overly PS-accumulating and cause phototoxicity [15]. PDT is made as effective as possible by combining targeted cell death with therapeutic drugs. This review describes how to find possible medications that are harmful to mitochondria in a variety of biological components and investigate the toxicological or pharmacological mechanisms behind them. It also emphasizes how PDT and synergistic techniques can work together to create novel approaches for cancer treatment.

## 2. Photodynamic Therapy

A breakthrough treatment called PDT produces singlet oxygen (S^0^) that is deadly to cancer cells by transferring photoelectrons to surrounding oxygen molecules using laser energy at a certain wavelength [16]. As a result, it is a treatment strategy that works well for epidermoid carcinomas and has the advantages of high spatiotemporal selectivity and low invasiveness [17]. Apart from eliminating cancer cells directly, PDT triggers an inflammatory reaction called immunogenic cell death, which encourages the production of tumor-associated antigens from the residuals of cancer cells [18]. However, much of the inadequate immune response under PDT is due to the immunosuppressive effect of the tumor microenvironment (TME) [19]. Furthermore, the TME’s condensed tumor extracellular matrix limits or impedes the efficient diffusion of oxygen and therapeutic chemicals, which lessens the impact of PDT [20]. Consequently, when used in conjunction with other form of treatment, PDT has a limited therapeutic effect on distal and metastatic cancers [21].

### 2.1. The Mechanism of Photodynamic Therapy

**Figure 1:**
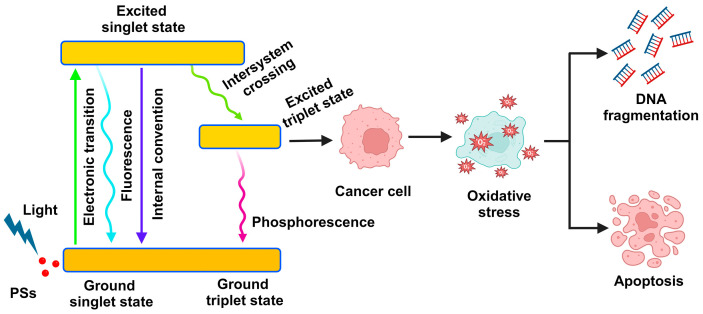
The mechanism of photodynamic therapy (PDT). Light absorption allows the photocatalyst to transition from a singlet energy state (S^0^) to an excited singlet state (S^1^) when exposed to light with a wavelength corresponding to the photothermal absorption (PS). The stored energy in the PS decays to a useful state, the excited triplet state (T^1^), but some of the energy is dissipated as an emission quantum. Cancer cells produce reactive oxygen species due to PDT, using light, oxygen, and PS to induce apoptosis. Meaning of the different colored arrows: green—Electronic transition, cyan—Fluorescence, blue—Internal convention, green—Intersystem crossing, purple—Phosphorescence.

To treat cancer, molecules known as PS transfer light energy to the lit area and alter the chemical structures nearby. A cancer cell may undergo irreversible alteration when the changes are great enough, which ultimately leads to the cell’s death [22]. There are two primary phases to the photodynamic reaction, and each is heavily reliant on oxygen molecules present within the cells. In the first phase of both strategies, the PS remains in its excited state (S^1^) until it either reverts to the ground state or undergoes additional photochemical processes inside the cell. The PS transitions from the lowest energy state S^0^ to the S^1^ state because of light absorption. The part of the energy that remains is released as a luminous particle as a PS molecule moves toward the applicable therapeutic triplet state (T^1^) (Figure 1) [23]. Protons can absorb energy from the PS in the triplet state T^1^ by activating their surroundings. When protons or electrons are transferred from PS to malignant tissue through the T^1^ state, an ionic process involving the PS produces reactive chemicals and substrates [24]. The superoxide anion radical (O_2_^•−^), the precursor to ROS, is first formed when highly reactive, excited electrons meet oxygen molecules. Through the following pathways, this initial ROS synthesis can encourage the generation of further ROS by cells. This process sets off a series of events that causes oxidative stress and ultimately causes cancer cells to die [25]. Increased levels of ROS in the photosensitive zone cause photodamage to lipids, proteins, and other molecules. This ultimately results in apoptosis or necrosis of tumor cells, which in turn causes cellular death [1].

### 2.2. Photosensitizers

Photosensitizers are essential to PDT’s efficacy. The best PS for PDT are ideally versatile, amphiphilic substances that work well in biological environments. These PS ought to have a long triplet lifespan, high molar absorption of long wavelength light, and an exceptional quantum yield for ^1^O_2_. They should also be non-toxic when exposed to no light [26]. In PDT research, numerous PS have undergone assessment both in vivo and in vitro; however, only a limited selection has shown optimal characteristics. Consequently, recent studies have focused on developing and assessing the effectiveness of new PS [24]. During PDT, harmless PS are administered systemically, capable of accumulating within cells and inducing cytotoxicity. Multiple studies have indicated the utilization of various substances as PS in PDT [27].

#### Types of Photosensitizers

In PDT, the multiple types of PS are utilized according to their progression, and they are further categorized into first, second, and third generation PS [28]. Hematoporphyrin gave rise to the first group of substances employed as PS in PDT. Second-generation substances such as synthetic photosensitizers, are also in use today. Third-generation drugs combine the strong efficacy of second-generation chemicals with an increased affinity for tumors, resulting in less harm to surrounding tissues [29]. Over time, various substances with photosensitizing characteristics have been identified or produced. The hunt for photosensitizers that selectively accumulate in neoplastic tissues and do not produce post-therapy adverse effects, such as cytotoxicity or mutagenicity, continues [30]. Scientists have created novel PS with enhanced biological characteristics and near infrared absorbances, which accumulate in neoplastic tissue and produce high quantum yields of ROS [31].

### 2.3. Light Source

Photodynamic therapy has utilized several light sources, including lasers, incandescent light, and LEDs [32]. To irradiate a larger tissue region, laser light sources can be used but are often expensive and need the use of an optical system to widen the light beam. Optical fibers can be used to specify the light wavelength for tissue irradiation with non-laser sources. In contrast, conventional lighting may have heat implications that must be avoided in PDT. Finally, PDT has used LEDs as lighting sources. LEDs are more widely available in flexible arrays, less expensive, less risky, and thermally nondestructive [33]. The therapeutic success of PDT is strongly dependent on the accuracy with which light is given to the target tissue, and its dose, which translates into light fluence, light fluence rate, light exposure time, and light delivery method, which are very critical components of PDT [24]. Light intensity, defined in J/cm^2^, is the sum of exposed light energy over a sectional area of an irradiation spot. Light intensity rate, measured in W/cm^2^, is the incident energy per second over a section of the irradiated site [34]. Lasers are commonly used in superficial and interstitial PDT procedures. One defining property of the lasers is the coherent creation of monochromatic light with a narrow bandwidth. Lasers give high optical power and an adjustable wavelength to match certain PS [35].

### 2.4. Drawbacks of PDT

During interaction with light sources in the days or hours following PDT, it is common to see pain and local skin reactions such as inflammation, swelling, peeling, or pustules, often in conjunction. Inflammatory pustular dermatosis on the forehead, discomfort from the contact, and hives are less common skin diseases. PDT has a significant impact on immunity, a fast onset, and may have long-term effects on the cancer growth connected with the treatment [36]. Scarring and hyperpigmentation are relatively infrequent side effects of PDT. Although it is not extensively described in the literature, hypopigmentation, induced by phototoxic damage to melanocytes, can occur [37]. Two cases of post-PDT bullous pemphigoid arthritis were described; one case was only affected at the PDT-treated sites by Bowen’s disease, while the other case also implicated other places [38]. There is no documented pathogenetic mechanism yet, however Wolf’s isotope reaction has been suggested as a possible explanation [36]. The most alarming of PDT’s potential side effects is its tendency to cause or stimulate the development of skin cancer. Several cases show that basal cell carcinoma, keratoacanthoma, and severe squamous cell cancer have formed after PDT treatment [39]. While some researchers claim that PDT does not directly create DNA mutations, others have shown that the ROS produced by PDT photosensitization can cause DNA damage and oncogenic activation [40].

### 2.5. Challenges in PDT Treatment

The development and improvement of PS still require additional work. Several concerns still need to be fixed before it can be used in the clinic going forward. Right now, several novel PS and PDT-based creative therapies are greatly improving PDT’s safety and effectiveness in the treatment of cancer [24]. Every therapy modality has advantages and disadvantages of its own. For example, in a combination chemotherapy-PDT system, the optimal results of cancer treatment depend on their appropriate partition ratio between chemotherapy and phototherapy. Increasing therapeutic agent targeting is essential to boost the efficacy of combination therapy for an antibody-drug system [1]. Due to several problems, photodynamic treatment is now only used in limited settings in clinics for a variety of purposes. The most significant obstacles to PDT are the unfavorable distribution of PS following intravenous administration, light attenuation through tissues that can lead to partial light delivery to the tumor area, hypoxia in the tumor environment that reduces the amount of oxygen available for PDT, partial or temporary disruption of the tumor vasculature following PDT and subsequent angiogenesis or vessel repair, and partial tumor destruction following PDT followed by tumor relapse or insufficient PDT-mediated stimulation of immunological responses against tumors [41].

## 3. Targeted Drug Delivery Systems

### 3.1. Drug Delivery in Cancer Targets Mitochondria

The rationale for developing mitochondria-targeted drug delivery strategies in cancer therapy stems from the unique properties of cancer cells as well as the limitations of conventional non-invasive drug therapies and biological processes. Abnormal malignant activity is a hallmark of cancer, making these cells and tissues important targets for major therapeutic interventions [42]. However, due to the double membranes and their highly efficient transport systems, the efficient delivery of drugs into mitochondria poses challenges that require new solutions. Cancer cells increase their activity and need for nutrients and required components, a feature that can be utilized to apply specific drugs [43]. Several methods have been developed to exploit important processes. The chemical conjugation of drugs with lipophilic cations, such as triphenyl phosphonium, exploits the poor solubility of the compounds, allowing the drugs to accumulate in solutes. This method is useful because it allows for the more efficient breakdown of drugs in the mitochondria, allowing them to be used in a variety of therapeutic approaches, from small molecules to peptides [44].

Mitochondria-targeted drug delivery holds the promise of increased therapeutic efficacy while minimizing the side effects associated with therapeutic treatment. Conventional chemotherapy regimens are nonselective, cause toxicity to healthy tissues, and limit the tolerated dose. Targeting therapeutic molecules to the mitochondria of cancer cells increases the drug efficacy at the target site, increases therapeutic efficacy, and reduces toxicity in the body [45]. Cancer cells often develop resistance to conventional therapies through a variety of mechanisms, including drug pumps, DNA repair mechanisms, and escape from apoptotic pathways. Targeting mitochondria, a critical component of apoptotic pathways, requires utilizing drug-resistant compounds that disrupt mitochondrial function [46]. Targeting drugs to mitochondria can activate apoptotic pathways, thereby activating the functional signaling pathways of the drug target and cellular components. Nano-combination therapy may interact with other anticancer drugs. For example, the increased levels of intracellular ROS caused by exposure to certain drugs can exacerbate radiation-induced injury, which depends on ROS-mediated DNA damage [47]. Similarly, immune checkpoint inhibitors can promote oncogenesis by regulating the immune response of cancer cells through chemokine-mediated signaling pathways [44].

### 3.2. Drug Targeting Mitochondrial DNA

Mitochondrial DNA (mtDNA) is a key biomarker of mitochondrial function. It encodes 37 coding genes, including 13 key subunits of the oxidative phosphorylation (OXPHOS) complex proteins and RNAs necessary for protein synthesis [48]. Mitochondrial DNA is essential for metabolic activity and cellular function. Mitochondrial DNA mutations and copy number changes were discovered to be intimately associated with the acceleration of tumor formation [49]. Mitochondrial dysfunction alters the energy metabolism and electron transport chain (ETC) system of malignant cells, resulting in changes in the mtDNA, which controls normal mitochondrial activity [50]. Genotoxic medicines cause a shift in mtDNA by regulating energy metabolism and mitochondrial oxidative phosphorylation, producing mitochondrial dependence and resistance to chemotherapy [51]. One study revealed that mtDNA plays a crucial and major part in tumor development, and an appropriate method would be to produce mitochondria-specific targeted medications as a line of defense against cancer [52]. Despite their importance in tumor development, mtDNA targeted treatments have recently emerged as viable cancer treatment possibilities [53]. The use of immunotherapy by inducing mtDNA release, targeted therapy by creating mitochondria-targeted complexes, and the combination of chemotherapy and PDT have significantly encouraged the progress of anticancer drugs targeting mtDNA [53]. Many bioactive compounds are now being tested in clinical trials to target mitochondria. Targeted medication delivery to mitochondria improves therapeutic efficacy while reducing harmful effects on non-malignant cells. Several medications have been used to target the function, structure, and metabolic activity of mtDNA to trigger cell death in cancer cells [52].

### 3.3. Mitochondrial Delivery by Nanoparticles

Nanoparticles (NPs) have become promising tools for delivering therapeutic drugs to specific cellular compartments, such as the mitochondria of cancer cells. Their versatile structures, high surface area, and surface functionality make them an ideal platform for the development of drug delivery and targeting [54]. The various types of NPs, including organic and inorganic nanoparticles, have been investigated for their ability to improve mitochondrial drug delivery and improve cancer treatment outcomes. Gold nanoparticles (AuNPs) have attracted much attention due to their unique properties and potential applications in cancer therapy [55]. Functionalized AuNPs can be induced to selectively accumulate in mitochondria based on their size, morphology, and morphological changes. The plasmonic properties of AuNPs allow for local near-infrared excitation, which provides a general advantage for drug delivery and photothermal therapy [56]. In addition, AuNPs can be loaded with functional molecules or conjugated with functional ligands, enabling the development of stable and multifunctional therapeutic strategies. Quantum nanoparticles (QDs), NPs with different morphologies, have emerged as promising biosensors for targeting and detecting cancer cells. QDs can be engineered to emit light in the near-infrared region, allowing deeper tissue penetration and improved imaging capabilities [57]. When functionalized with tumor-targeting ligands, QDs can selectively accumulate in the tumor, providing precise insights into intracellular processes and enabling the monitoring of drug delivery [44]. Mesoporous silica nanoparticles (MSNPs) provide a unique platform for drug encapsulation and delivery to specific cellular compartments. MSNPs have a large surface area and an adjustable pore diameter, which allows for efficient absorption and precise release [58].

### 3.4. Mitochondrial Delivery by Lipid-Based Nanocarriers

Lipid-based nanocarriers have emerged as versatile and effective vehicles for drug delivery to the mitochondria of cancer cells. These carriers exploit the permeability of lipids to cells, allowing the effective encapsulation, storage, and control of the drugs [59]. Lipid-based NPs can be engineered to selectively accumulate in mitochondria, offering many advantages for improving drug delivery and pharmacokinetics. Liposomes, NP-encapsulated membrane vesicles, were one of the first and most widely studied lipid bilayers for drug delivery. Their biocompatibility, unique physical properties, and ability to incorporate various types of hydrophilic and hydrophobic compounds make them attractive adsorbents [60]. To facilitate tumor targeting, liposomes can be functionalized with effective lipids or ligands, such as peptides or antibodies, that mediate binding to the malignant matrix and facilitate their emergence [61]. Solid lipid NPs are lipid-based materials that provide greater stability and negative control than traditional liposomes. They are composed of lipids that are solid or soluble at room temperature, thus protecting the encapsulated drugs and allowing them to be released [62].

### 3.5. Mitochondrial Delivery by Peptides

Peptide-based delivery has been developed as a highly efficient platform for delivering therapeutic agents to the surface of cancer cells [42]. Peptides, composed of amino acids, can be modified to have an affinity for specific parts of the body, allowing for selective and precise drug delivery [43]. These peptides can be engineered to attach directly to cell membrane or even reach the inner membrane, providing a sophisticated and flexible approach to cellular drug delivery [44]. Membrane-permeating peptides (MPPs) are small peptides with the unique ability to pass through cell membranes. MPPs often include positively charged amino acids, allowing them to interact with negatively charged amino acids and facilitate exchange. These peptides improve drug distribution and prevent undesired side effects by conjugating drugs to MPPs [45]. Peptides can be designed to bind to specific proteins or receptors on the surface of the protein. These targeting peptides take advantage of the intracellular import processes that allow them to selectively accumulate within the cell. By combining inhibitory peptides with targeted drugs, researchers can achieve cargo encapsulation, increase the drug’s targeting index, and reduce damage to off-target cellular components [46]. Implantation of an intravenous system poses additional limitations due to its inaccessibility. However, this allows for isolated peptides that stimulate the release of molecules into the extracellular matrix [47].

### 3.6. Challenges in Mitochondrial Drug Targeting

Although the development of mitochondria-targeted drug delivery platforms has great potential to improve cancer therapy, various challenges and considerations must be addressed before these approaches can be successfully applied from the bench-top. Overcoming these challenges is critical to realizing the full potential of mitochondria-targeted therapies and their clinical benefits. For therapeutic drugs to enter the mitochondria properly, barriers to cellular uptake and drug trafficking must be overcome. Cancer cells have dynamic and heterogeneous characteristics that affect nanoparticle uptake, such as cell size, morphology, and receptor expression [59]. Furthermore, obtaining multiple dosing regimens requires navigating the complex mechanisms of intracellular and intestinal absorption, which may vary depending on the type of NP used. The main challenges are to develop strategies to increase NP uptake and maintain mitochondrial fitness [44].

The heterogeneity of cancer cells makes it difficult to deliver targeted and co-administered drugs. Differences in membrane potential, mass, and morphology between cancer types, as well as within a single tumor, complicate the development of targeted therapies that account for this heterogeneity [63]. Adaptation of the drug delivery systems to accommodate this disparity while maintaining drug availability is an important consideration [60]. For mitochondrial drug delivery to be effective, therapeutic chemicals must be precisely targeted to delivery sites and released under controlled conditions once they reach the target. Efficient drug transport, uptake, and degradation are critical to maintaining mitochondrial homeostasis [61]. The choice of transporters, surface modifications, and release conditions must be carefully considered to ensure proper delivery and targeting during mitochondrial transport [44].

## 4. Drug-Induced Mitochondrial Toxicity

About 95% of the adenosine triphosphate (ATP) in cells is generated by mitochondria, the so-called “energy powerhouse” of a cell, by OXPHOS. Additionally necessary for maintaining life and determining cell fate are the mitochondria [64]. The numerous functions of mitochondria, such as ATP synthesis, cell death, immune system, signal transmission, and energy metabolism are regulated by the cell in normal and abnormal circumstances [65]. During the development of a disease, pathogenic reactions may alter mitochondrial function and dynamics. OXPHOS is frequently hindered by mitochondrial malfunction, which raises glycolysis and causes deadly lactate buildup in the serum [66]. Drug-induced mitochondrial cytotoxicity is well-recognized to harm the liver, skeletal muscles, kidneys, the heart, and the central nervous system. Anti-diabetic and cholesterol-lowering drugs, anti-depressants, painkillers, specific antibiotics, and anti-cancer therapies are all linked to mitochondrial toxicity [67]. Numerous medications have been removed from distribution by the US Food and Drug Administration (FDA), since the late 1990s. Due to their hepatic injury or neurotoxic effects, a number of these drugs have been linked to issues with mitochondria [68].

The toxicity of drugs towards mitochondria has already been researched for more than 50 years. Numerous pathways of mitochondrial toxicity have been reported, such as the inhibition of OXPHOS, disruption of intracellular signaling, elevated ROS production, induction of the mitochondrial membrane potential (Δψm), decreases in the production of fatty acids or the tricarboxylic acid cycle (TCA), decreased production of FADH2 and NADH, impairment of cognitive function, and interference with mitochondrial DNA (mtDNA) replication [69,70]. However, the processes underlying mitochondrial toxicity and dysfunction are currently not fully known. Substantial advances were made in the last 10 years with the advent of various viable epithelium and in vitro models for preclinical research [71]. Most of these findings were reached through research using cell lines and isolated mitochondria [71]. Newly created cell models cultured in galactose can more precisely and sensitively identify possible drug-induced mitochondrial toxicity than previous work in model systems grown in high glucose, which were non-responsive to such toxic effects because they switched to glucose metabolism for energy production [72]. Utilizing oxygen sensors that are soluble and solid, drugs can now be examined in 96- and 384-well formats for the possibility of inducing mitochondrial toxicity [73]. In cancer cells, drug-induced mitochondrial toxicity often occurs through mechanisms such as disrupting mitochondrial membrane potential leading to reduced ATP synthesis, increased ROS formation, and the activation of apoptotic pathways [74]. Furthermore, some chemotherapeutics can directly target mtDNA or proteins, causing metabolic malfunction and cell death. Understanding these pathways can assist in creating strategies to reduce toxicity while maintaining therapeutic efficacy [53].

### 4.1. Mitochondria-Independent Chemotherapeutics

Some chemotherapeutics induce mitochondrial damage through non-mitochondrial targeting, while agents such as IACS-010759 (IACS) directly inhibit the mitochondrial ETC, exhibiting potential as agents against cancer [75]. IACS selectively inhibits complex I, demonstrating antitumor effects in glycolysis-deficient tumor cells with minimal toxicity to normal cells at acceptable doses, according to a recent study [76]. IACS showed a narrow therapeutic index in another study, exhibiting toxicities at limiting doses, such as increased blood lactate and neurotoxic effects, impeding target exposure maintenance [77]. IACS, an oral inhibitor targeting mitochondrial complex I, exhibited strong responses in preclinical tumor models, justifying clinical testing with good tolerance and initial antitumor activity [78]. A different study indicated that the newly developed mitochondrial complex I inhibitor, IACS, exhibited preclinical efficacy in NOTCH1-mutated T-ALL, suggesting potential improvements in patient outcomes [79]. More recently, it was shown that the killer lymphocyte protease granzyme A substrate is an iron-sulfur component called NDUFS3. Targeted cell death caused by the caspase-independent cleavage of NDUFS3 at the mitochondrial matrix occurred when the membrane potential was disrupted, and ATP production was inhibited [80]. Viral RNA must target complex I to sustain the potential of the mitochondrial membrane and produce ATP, which is necessary for the viral propagation cycle [81]. Transgenic mice, whose ribozymes targeted the complex I subunit NDUFA1, showed a loss of complex I activity. Degeneration of the optic nerve and elevated ROS levels in retinal ganglion cells were seen in these mice [82].

### 4.2. Effects of PDT on the Mitochondrial Membrane Potential

Photodynamic therapy alters Δψm, a key indicator of mitochondrial activity and energy generation. During PDT, ROS buildup can compromise mitochondrial membrane integrity, resulting in a reduction in Δψm [83]. This disruption inhibits ATP generation and can trigger apoptotic pathways by releasing pro-apoptotic proteins such as cyt c [84]. Evaluating Δψm alterations after PDT can show treatment efficacy and the amount of mitochondrial dysfunction in targeted cells [85]. Understanding these dynamics is critical for refining PDT methods and improving therapeutic outcomes while limiting harm to nearby healthy tissues. Thus, the relation between PDT and Δψm is an important topic of study in cancer therapy.

## 5. Mitochondria as a Potential Cancer Therapeutic Target

### 5.1. Apoptosis Regulation

The reorganization of several pathways within cancerous cells consistently reveals certain weaknesses that may be leveraged in treatment approaches. Nevertheless, the development of such therapeutic techniques is limited by the variety of tumors and the presence of compensatory pathways. Various drugs that target different parts of the mitochondrial metabolism have been proposed thus far as possible cancer therapies (Table 1). Metformin can block complex I of the ETC in mitochondria, which is related to bioenergetics. This lowers oxygen consumption, tampers with the NAD+/NADH ratio, and decreases ATP synthesis [86]. The decreased TCA activity and the resulting severe energetic stress cause AMPK activation, autophagy, mTOR pathway inhibition, and reduced TCA activity [87]. Abundant evidence suggests that targeting PI3K/Akt/mTOR-mediated autophagy could enhance chemosensitivity in tumor cells and mitigate drug resistance [88]. Some inhibitors, currently in clinical trials, have not succeeded in enhancing patient outcomes, primarily attributed to their influence on the mitochondrial reprogramming in cellular bioenergetics and trafficking [89]. As the disrupted equilibrium between ROS synthesis and detoxification serves as the fundamental biochemical pathway driving the abnormal proliferation and survival of tumor cells across various cancer types, the targeting of mitochondrial ROS (mROS) signaling is considered to have promising therapeutic potential. Because vitamin K3 causes ovarian cancer cells to produce more ROS, which results in apoptosis, it possesses the potential for development as an anticancer pharmaceutical [90]. Conversely, many cancer cells benefit from mROS production due to its impact on redox signaling; as a result, sophisticated approaches that permit the precise control of mROS must be developed. Today, a variety of therapeutic methods that influence ER Ca^2+^ release is used to modify mitochondrial Ca^2+^ transport [91]. These include mipsagargin and photodynamic therapy, which induce apoptosis by stimulating mitochondrial Ca^2+^ excess and ER Ca^2+^ depletion [92].

### 5.2. Metabolic Rewiring

Several methods, such as boosting glucose absorption and glycolysis, and switching to glutamine usage, are used by cancer cells to counteract the effects of antitumor drugs. Due to this, the glycolytic inhibitor 2-deoxyglucose (2-DG) and metformin were suggested as a combined therapy for cancer. The negative effects of high-dose treatment with a single drug are reduced by this synergistic combination’s ability to greatly decrease ATP storage and inhibit the activation of the proliferative signaling pathway [109]. 2-DG has a limited therapeutic effect on various cancers, but when paired with chemotherapy or radiation, it can have a synergistic antitumor effect. 2-DG, a glucose analog, competes with glucose for hexokinase binding, raises oxidative stress, triggers autophagy, and raises apoptosis, which inhibits the proliferation of cancer cells [110,111]. Its modest anticancer effectiveness, however, might be caused by enhanced autophagy, which keeps cancer cells alive. To effectively target different mitochondrial pathways, this is important. The glycolytic inhibitor 2-fluoro-deoxyglucose (2-FDG) has recently been shown to be more effective than 2-DG. However, recent research suggests that the cytotoxicity of these two analogs is influenced by the environment in which tumor cells thrive [112]. It is interesting to note that several brand-new CI inhibitors, such tamoxifen with mitochondrial targeting and BAY 872243, have been shown to cause cancer cell death and lower cell growth [93]. Another drug designed to target mitochondria, vitamin E succinate, inhibits complex I and, more severely, CII, increasing ROS generation and causing breast cancer cells to die [95]. One class of CII inhibitors that impacts glutamine metabolism and the TCA cycle in melanoma cell lines is lopinidamine. It has been used to improve the efficacy and general response to cancer therapy when combined with other chemotherapy medications [96]. Recent work by Brummer et al. demonstrates that the RAF inhibitor vemurafenib can sensitize human melanoma cells to diclofenac and lumiracoxib, two nonsteroidal anti-inflammatory medications, by enhancing its anti-glycolytic effects and impeding metabolic reprogramming towards OXPHOS [97]. In a related study, venetoclax and WEHI-539, two BCL-2 and BCL-XL inhibitors were coupled with 2-DG to lower cellular bioenergetics and eliminate the ability of breast cancer cells to become clonogenic [98]. Another prescription drug known as VLX600 is an ETC inhibitor that, especially in low-glucose settings, can impair mitochondrial bioenergetics and halt tumor growth. Numerous studies have shown the potential influence of lower mitochondrial protein translation and stability on mitochondrial bioenergetic capacities, in addition to the direct impacts of ETC inhibitors [98]. In fact, gamitrinib, which was designed to concentrate in mitochondria, was shown by Chae and colleagues to block HSP90 and TRAP-1 ATPase activity, which reduced tumor cell proliferation [99]. CPI-613, a brand-new lipoic acid derivative, is one of many inhibitors of the mitochondrial metabolism-related enzymes that specifically target mitochondrial metabolism in tumor cells. Pyruvate dehydrogenase (PDH) is hyperphosphorylated and AMPK is activated because of CPI-613’s inhibition of mitochondrial respiration through PDH and α-KGDH inactivation [100].

### 5.3. Therapeutic Strategies

Patients with advanced hematological malignancies who received CPI-613 alone or in combination with a high dose of the chemotherapy medicines cytarabine and mitoxantrone saw encouraging outcomes in phase I research [101]. Additionally, CPI-613 was able to slow the growth of tumors when combined with either chemotherapy or chloroquine. These intriguing data emphasize the significance of simultaneously addressing many routes simultaneously [113]. Only two of the mutant IDH inhibitors examined in clinical trials enasidenib and ivosidenib have been authorized for use in the treatment of refractory AML [102]. In phase I studies, the safety and tolerance of various mutant IDH inhibitors have also been investigated in glioma, cholangiocarcinoma, and chondrosarcoma, among other cancer types. Alternative strategies are focusing on pyruvate dehydrogenase complex and pyruvate dehydrogenase kinase, two crucial enzymes in glucose metabolism that are usually altered during carcinogenesis [114]. It has been demonstrated that DCA, a PDK inhibitor that also acts as a PDC activator, inhibits the growth of cancer cells. However, due to its nonspecific activity, lack of potency, and high dose requirements, its therapeutic applicability is limited [115]. DCA derivatives were created to specifically target the pyruvate-binding area and lessen the toxicity of DCA. Pyruvate, which is overexpressed in several cancer types and is converted to lactate by lactate dehydrogenase A, is another potential target for anticancer treatment [116]. Numerous inhibitors with distinct mechanisms of action have been created, but only one naturally occurring phenol, gossypol, that competes with NADH, has been studied in clinical trials [103]. Another potent anticancer strategy would be to target the mitochondria’s function, which is vital for cellular communication and signal transmission. There is strong evidence that autophagy inhibitors increase the effectiveness of chemotherapy, which is consistent with the hypothesis that autophagy actively contributes to drug resistance. For instance, CQ and sorafenib work together effectively to treat hepatocellular cancer [117]. In patients with colorectal cancer, tioconazole enhances the efficacy of doxorubicin by inhibiting autophagy through its targeting of ATG4B [104]. Verteporfin inhibits the production of autophagosomes and makes pancreatic ductal adenocarcinoma more sensitive to gemcitabine [105]. Recently developed novel mitochondrial calcium uniporter (MCU) complex inhibitors include ruthenium complex Ru265; mitoxantrone; pixantrone; and DS16570511 [107]. Nevertheless, their biological functions may not solely depend on MCU inhibition, and additional research may clarify how the MCU complex and mitochondrial Ca^2+^ contribute to the development of tumors [3]. The main source of carbon for the TCA cycle and its intermediates is glutamine. Reducing the energy of cancer cells may be achieved by focusing on glutamine catabolism. In addition to blocking glutamine catabolism, the GLS inhibitors substance 968 and bis-2-(5-phenylacetamido-1,2,4-thiadiazol-2-yl)ethyl sulfide delay the development of cancer [108]. These aspects—the variety of treatment targets and their cellular adaptability—highlight the intricate and contentious role that mitochondria play in cancer cell metabolism.

## 6. Mitochondrial Drug Delivery Mechanisms in Cancer

The application of mitochondria-targeted drug delivery strategies in cancer therapy offers a wide range of strategies that can improve therapeutic efficacy. These approaches exploit the unique properties of the cancer cell to interfere with key processes involved in cancer development, progression, and drug resistance [118]. Intracellular ROS plays a complex role in cancer. Although the excessive production of ROS can damage cellular components including DNA, proteins, and lipids, dysregulated mitochondrial ROS can also activate apoptotic pathways leading to cell death [55]. Antibiotics that increase intracellular ROS use this mechanism to target cancer cells. By taking advantage of the increased mitochondrial activity and nutrients required by cancer cells, these drugs can mobilize the redox balance against inflammatory stress, thereby inducing apoptosis and inhibiting tumor growth [56]. For example, the platinum-based drugs (cisplatin, doxorubicin, etoposide) have this mechanism. Mitochondria-targeted therapies are a new approach to cancer treatment. These drugs are designed to target the mitochondria, where they can interfere with important processes necessary for cancer cells to survive [57]. Oral gastrointestinal peptides are a well-known family of pharmaceutical compounds that can enter the gastrointestinal tract and disrupt gastrointestinal function. Another example are mitocans, which are synthesized on target cells and cause cell death by interfering with the inflammatory response [44]. These mitochondrial inhibitors may enhance therapeutic efficacy by directly disrupting mitochondrial function, making cancer cells more resistant to conventional therapies. PDT uses PS that can produce harmful ROS when exposed to light at certain temperatures [44]. Mitochondrial activation by PS contributes to mitochondrial ROS production. This strategy takes advantage of the inherent ROS generation capabilities of mitochondria to promote oxidative stress and PDT-induced apoptotic pathways. Pharmaceutical PS, such as porphyrin- and chlorin-based preparations, have shown promising results in preclinical studies, promising additional benefits [58].

### 6.1. Photodynamic Therapy-Induced Mitochondrial Toxicity

Photosensitizers have certain drawbacks, including the fact that prolonged or increased exposure to activating light can have unfavorable effects [119]. The creation of innovative PS that enhance therapeutic efficacy without imposing undesired constraints can address this limitation. The efficiency with which a PS uses light energy to convert molecular oxygen to S^0^, known as the singlet oxygen quantum yield, is the most significant parameter used to compare the efficacy of various PS [120]. The best PS for therapy are those with the highest quantum yield because S^0^ is a strong oxidant that can directly harm cells. However, the toxicity of PDT is primarily based on the PDT agent’s subcellular location; the literature has revealed no clear association between S^0^ generation and cell death [121]. The aiming time limit should be small since S^0^ is extremely reactive and easily quenched. Furthermore, the location of its oxidative damage will most likely be the same as its place of creation [122]. The drugs for photodynamic treatment must concentrate in the organelles that contain the produced ROS effectively, and its harmful effects would need to be perceived sensitively to overcome this limitation [123]. Because the mitochondria contain high concentrations of oxygen and even the small amounts of S^0^ created are more hazardous than the huge amounts produced in the nucleus, it would be advantageous to target the mitochondria. Techniques to improve the effectiveness of mitochondrial targeting have been developed [124,125]. Furthermore, verteporfin, an FDA-approved medication that is widely used to treat wet age-related macular degeneration, has a longer absorption wavelength than other mitochondria-targeting PS, making it perfect for deeper tissues [126]. Consequently, there appears to be potential to create PS for PDT that target mitochondria.

#### 6.1.1. Apoptosis

**Figure 2:**
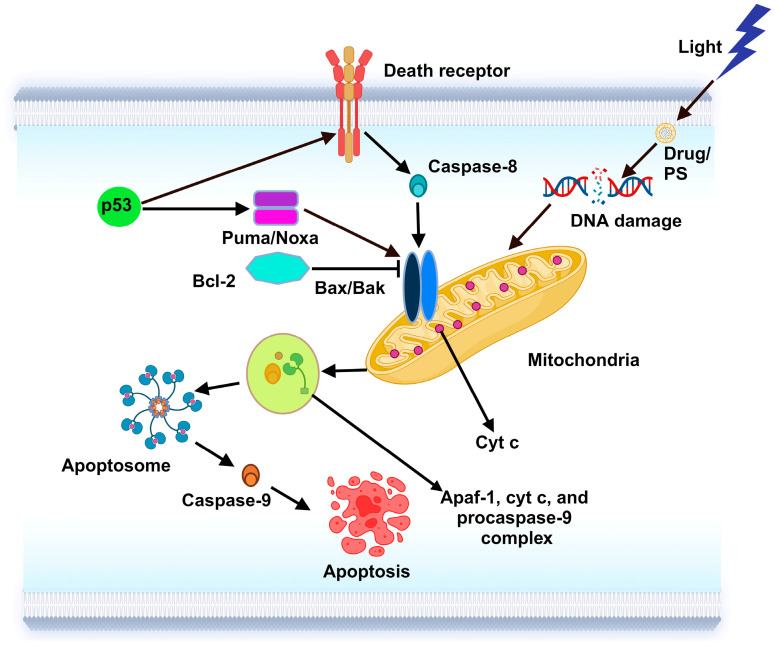
The mechanism of apoptosis. The primary stage of the apoptotic process is driven by mitochondria, which permits a variety of apoptogens to approach the cytosol and initiate procaspases. When cytochrome c (cyt c) is released, a multiprotein complex called the apoptosome is formed. This component cleaves procaspase-9 to activate caspase-9, which in turn stimulates the production of other inhibitors of apoptosis. To release cyt c and complex 1 (Apaf-1) from mitochondria and form the procaspase-9 complex that activates caspase-9, the tumor suppressor protein (p53) is also involved in the emergence of the mitochondrial apoptosome. In contrast, Bcl-2 can bind to Bax/Bak and sequester BH3 proteins, which inhibits this process of cell death and promotes cell survival.

Various methods are used to start the apoptosis pathways following PDT-induced organelle breakdown [24]. Apoptotic cell death with direct damage happens when the PS is in the mitochondria, resulting in Bcl-2 breakdown and the release of cyt c [127]. The apoptotic execution machinery is activated by mitochondria in mammalian cells [128]. Numerous factors can trigger mitochondria-related apoptosis, including ischemia/reperfusion, nutritional deprivation, oxidative stress, elevated Ca^2+^ levels, chemotherapeutic drugs, and certain cancer therapies [129]. The outer mitochondrial membranes (OMM) become permeable, allowing different apoptogens to enter the cytosol and trigger procaspases. This is the initial stage of the mitochondria-driven apoptotic process. The BCL-2 proteins, a family of proteins classified into three subfamilies according to their roles and their Bcl-2 homology (BH) domains, tightly control the entire system. These proteins include the following: (i) pro-survival proteins such as Bclw, Mcl-1, Bcl-xL, and Bcl-2 itself; (ii) pro-cell death proteins such as Bax, Bak, and Bok (containing BH1-3); and (iii) pro-cell death proteins such as Bim, Bid, Puma, and Noxa (containing BH3 alone) [130]. When BH3 proteins physically attach to the OMM, they experience homo- or hetero-oligomerization because of the conformational activation of Bax and Bak. This critical stage induces apoptosis by activating cytosolic procaspases, which results in OMM permeabilization and mitochondrial apoptogen leakage [131]. The apoptosome, a multiprotein complex that cleaves procaspase-9 to activate caspase-9 and subsequently activates additional apoptotic effectors, is formed in response to the released cyt c [132]. By stimulating cyt c and apoptotic protease-activating factor 1 (Apaf-1) from mitochondria and causing them to form a complex with procaspase-9, which activates caspase-9, the tumor suppressor protein p53 also plays a role in the activation of the apoptosome (Figure 2) [133]. In contrast, Bcl-2 can bind to Bax/Bak and sequester BH3 proteins, which prevents this death process and encourages cell survival [134]. To effectively eliminate tumor cells, combination therapy targets these mitochondrial pathways by boosting intrinsic apoptosis through ROS-induced cyt c release, modifying extrinsic apoptosis by death receptor sensitization, and promoting necroptosis through mitochondrial dysfunction [135]. Combination therapy specifically targets mitochondrial toxicity in cancer cells, utilizing their altered metabolism and weakened antioxidant defenses, resulting in increased ROS and mitochondrial dysfunction while protecting healthy cells with more stable mitochondrial functions [136].

#### 6.1.2. Autophagy

**Figure 3:**
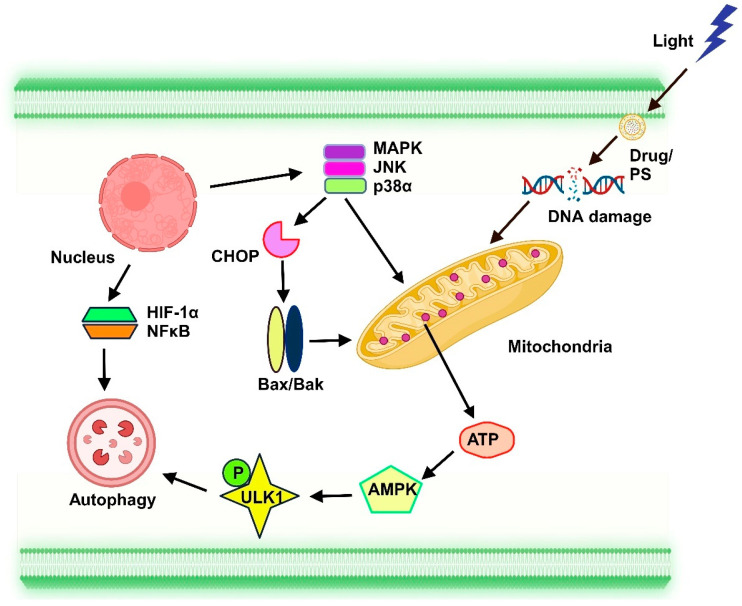
The mechanism of autophagy. The activation of the photosensitizer in the mitochondria results in a decrease in ATP synthesis and an increase in reactive oxygen species production. The energy-sensing AMPK activates ULK1, which starts autophagy. Photodynamic therapy can also activate NFκB, an autophagy mechanism involved in protein, lipid, and nucleotide production, to initiate lysosome formation and autophagy. Autophagy can be transcriptionally controlled by mitochondrial photooxidation via MAPK, CHOP, and HIF-1α.

The effects of PDT can cause apoptosis in cells or not, depending on the amount of light exposure and PS given [137]. The PS localized in the lysosome diminishes and eventually kills the cell when exposed to light. PDT generates enough ROS to induce apoptosis; if ROS is not produced, autophagy is activated to maintain cell survival. Increased Bcl-2 protein levels shield cells against PDT-induced phototoxicity [138]. Following PDT, resistant mouse cells expressed anti-apoptotic Bcl-2 protein more frequently, leading to non-apoptotic cell death [139]. Nevertheless, alternative studies suggest that the Bcl-2 protein prefers binding with Beclin-1, which suppresses the autophagic response responsible for PDT resistance [140]. The pro-survival process of autophagy is initiated when PDT-induced cell damage triggers certain cellular components to degrade and recycle (Figure 3) [141]. The PS produces ROS when it is activated in the mitochondria, which lowers the synthesis of ATP. This drop in ATP is detected by AMP-activated protein kinase (AMPK), which then uses autophagy-initiating kinase 1 to initiate autophagy and reprogram metabolism. Furthermore, through the hypoxia-inducible factor 1-alpha (HIF-1α)/VIMP1 and mitogen-activated protein kinase (MAPK)1/3 pathways, cytoplasmic photodamage in PDT enhances the activation of autophagy and the levels of nuclear factor kappa-light-chain-enhancer of activated B cells (NFkB) [142,143]. The energy-sensing AMPK triggers the activation of autophagy activating kinase 1 (ULK1), which starts autophagy. PDT can activate the autophagy machinery through NFkB and initiate lysosome formation and autophagy by promoting the synthesis of proteins, lipids, and nucleotides. Additionally, the transcriptional regulation of autophagy can be achieved through the photooxidation of mitochondria via the actions of HIF-1α, C/EBP homologous protein (CHOP), and MAPK [144]. Furthermore, conditions such as starvation and oxidative stress from ROS generated during PDT can encourage autophagy activation [144].

### 6.2. Nanoparticle-Based Delivery Mechanisms in Cancer

Nanocarrier-based drug delivery systems for chemotherapeutic medicines work effectively on numerous cancerous sites. The most popular drug delivery methods rely on organic and inorganic particles [145]. Micelles, liposomes, polymers, dendrimers, and nanogels are examples of the organic particles utilized in drug delivery applications. They feature diverse surface building elements that enable efficient endocytosis and loading [146]. NPs have multifunctional surface-modifying properties which stimulate cells to the tumor vasculature. The method of encapsulating chemotherapy therapies using a nano-scale device is the greatest option for reducing side effects and increasing drug bioavailability for cancer [147]. While developing technologies of systemic drug delivery via NPs hold promise for early cancer treatment, patients with metastatic cancer currently have few options. Technologies based on nanocarrier platforms are the most effective technique for precise site targeting in drug-resistant cancer cells [148].

## 7. Clinical Developments in PDT and Mitochondria Targeting in Cancer

Over the past 30 years, PDT has participated in clinical studies for a range of cancer types, with an emphasis on superficial tumors such cutaneous carcinoma, esophageal cancer, and oropharyngeal cancer [149]. Many preclinical cancer models have been employed since the mid-1950s to investigate PDT as a possible treatment; in the early 1990s, improved selectivity and specificity led to the acquisition of clinical approval for the treatment of cancer [150]. It has become clear that PDT’s noninvasiveness, safety, temporal and spatial selectivity, and the lack of extreme effectiveness are essential for clinical approval. However, PDT by itself has a limited therapeutic efficacy against various solid tumors that are deep or hypoxic due to its inherent limits and the clinical challenges involved with cancer therapy [151]. This claim is supported by the increasing integration of PDT-combined techniques in clinical trials for the treatment of non-small cell lung cancer, basal cell carcinoma, and other malignancies. Finnish institutions are currently using ALA nano formation-based PDT in clinical trials for skin cancer, including Tampere University and the University of Jyvaskyla. When it comes to PDT, the usage of the BF200 ALA gel (ClinicalTrials.gov: NCT01966120) demonstrated that, in contrast to 21.9% of patients who received a placebo nanoemulsion, 90.9% of receivers had an overall good patient response. Further details and other verified primary and secondary outcomes measurements can be found on the website https://www.clinicaltrials.gov/ (Accessed on 27 June 2024). On the other hand, nonserious adverse events including pain (96.36%), erythema (92.73%), pruritus (38.18%), scab (36.36%), exfoliation (30.91%), edema (21.82%), vesicles (10.91%), discomfort (9.09%), and discharge (5.45%), as well as serious adverse events including acute myocardial infarction (1.82%), femoral neck fracture (1.82%), bursitis (1.82%), and cardiovascular accidents (1.82%) [152]. The data above point to the inevitable adverse effects, and before using any therapeutic strategy, it is possible to thoroughly review it. Additionally, each patient receives a unique assessment, especially when therapy has an impact on their way of life [24].

Immunohistochemistry has become a promising tool in cancer research, providing new strategies for diagnosis, treatment, and prevention. Mitochondria, called the powerhouse of the cell, are responsible for generating cellular energy and regulating apoptosis. Mitochondrial dysfunction is a hallmark of cancer cells, which have high energy demands for rapid proliferation and survival. Therefore, increasing attention has been paid to its application against cancer [44]. One of the most promising clinical applications of mitochondrial dysfunction in cancer is imaging. Molecular approaches targeting specific mitochondrial function may help in early cancer detection and tumor localization [42]. Flow cytometry analysis measured cellular metabolism, redox status, and calcium levels, providing important information on the dynamics and potential solutions of cells scavenging normal tissues. This allows doctors to create individualized treatment programs based on the unique characteristics of individual patients. In addition, mitochondria-targeted therapies have shown promise in cancer therapy [43]. Researchers can find new cancer targets and treatments by studying the complex interactions between genes, molecules and cells. DNA mutations and transcriptional activation have been linked to cancer. Strategies to preserve mitochondrial integrity may hold the key to cancer prevention in high-risk populations [45]. Table 2 lists the chemicals and genes tested for specific transporters. The bench-to-front-line pathway for mitochondria-targeted cancer therapy involves extensive preclinical in vitro research and in vivo clinical trials, followed by further clinical trials.

## 8. Preclinical Studies: Cellular Assays and Animal Models

In vitro studies are often performed to evaluate the safety and efficacy of mitochondria-targeted drug delivery strategies in preclinical studies. Cancer cell lines with different phenotypes have been used to study NPs, subcellular localization, and changes in cell function [44]. This type of study revealed how mitochondria-targeted drugs affect cell survival, apoptosis, and apoptosis. Parameters such as membrane potential, ROS production, and intracellular DNA damage are commonly used to evaluate the efficacy of these drugs. Moving to in vitro models, animal studies have provided important information on the effects of drugs affecting mitochondria in vivo [44]. Xenograft models allow for the evaluation of therapeutic efficacy, tumor growth inhibition and immunogenicity [156]. NP distribution and mitochondrial localization can be monitored in vivo using imaging techniques such as positron emission spectroscopy or magnetic resonance imaging [157]. Animal studies have also investigated possible interactions between mitochondria-destroying drugs and other conventional treatments, such as chemotherapy or radiation [58]. Several mitochondria-targeted drugs have undergone clinical trials, providing encouraging data on their potential therapeutic benefits. Other prominent examples are the studies on the safety and efficacy of anticancer drugs. These studies often focus on outcomes such as overall survival, progression-free survival, and quality of life, providing important information about the treatment outcomes and potential limitations of these approaches in real-world clinical settings [44].

## 9. Conclusions and Future Perspectives

The combination of PDT with specific drug delivery systems has great potential for improving cancer treatment. Future research should concentrate on a few important areas to improve the efficacy and applicability of this combination strategy. Innovations in photodynamic agents, such as the development of novel PS, with improved light absorption capabilities and tissue penetration, could help to solve present PDT limits. Advances in targeted drug delivery methods, such as the creation of more complex NPs, and lipid-based and peptide-mediated mitochondrial targeting, may improve the specificity and efficiency of drug delivery to cancer cells. Furthermore, the use of real-time imaging tools to assess therapy responses and optimize treatment protocols is an important step toward personalized medicine. Addressing issues such as safety, distribution efficiency, resistance mechanisms, and cost is critical to the successful transition of these technologies from preclinical to clinical practice. Developing measures to reduce potential off-target effects and improve patient safety will be critical. Furthermore, looking at ways to cut prices and enhance accessibility can make these advanced medicines more affordable for general use. To summarize, the combination of PDT with targeted drug delivery systems represents a promising future in cancer treatment. By combining the characteristics of both modalities, this method has the potential to overcome current therapy constraints and provide more effective, individualized cancer drugs. As research advances and new technologies emerge, this combination strategy may open the way for considerable improvements in cancer treatment results and patient quality of life. Our future research should investigate how drug delivery methods combined with PDT can modulate the mitochondrial membrane potential heterogeneity in cancer cells through in vitro studies to improve therapeutic efficacy.

## Figures and Tables

**Table 1:** An overview of the drugs mentioned in this review and their mode of action.

Drug	Type of Cancer	Model	Mitochondria Function	Mechanism of Action	References
Metformin	Breast, prostate, melanoma, ovary, and lung	Clinical trials	Bioenergetic	Inhibition complex I	[86,87]
Vitamin K3	Ovary	Clinical trials	Signaling	Increasing generation of ROS	[90]
Mipsagargin (G-202)	Glioblastoma and liver	Clinical trials	Signaling	Mitochondrial Ca^2+^ transfer modulation	[92]
BAY 87-2243	Lung and prostate	Clinical trials	Bioenergetic	Inhibition complex I	[93]
IACS-010,759	Acute myeloid leukemia and chronic lymphocytic leukemia	Clinical trials	Bioenergetic	Inhibition complex I	[94]
MitoVES	Breast	Preclinical	Bioenergetic	Inhibition complex I and II	[95]
Lonidamine	Lung	Clinical trials	Bioenergetic	Inhibition complex II	[96]
Venetoclax and WEHI-539	Breast	Preclinical	Bioenergetic	Reducing bioenergetic	[97]
VLX600	Colon	Clinical trials	Bioenergetic	ETC inhibitor	[98]
Gamitrinib	Lung and prostate	Preclinical	Bioenergetic	Inhibition HSP90 and TRAP-1 activity	[99]
CPI-613	Pancreas and hematological cancers	Clinical trials	Bioenergetic	PDH and α-KGDH inhibitor	[100,101]
Enasidenib and Ivosidenib	Acute myeloid leukemia	Clinical trials	Bioenergetic	Mutant IDH inhibitors	[102]
Dichloroacetate	Lung and liver	Clinical trials	Bioenergetic	PDKs inhibitor	
Gossypol	Breast, brain, and prostate	Clinical trials	Bioenergetic	LDHA inhibitor and NADH competitor	[103]
Tioconazole	Colon	Clinical trials	Signaling	Blocking autophagy targeting ATG4B	[104]
Verteporfin	Pancreas	Clinical trials	Signaling	Blocking autophagosome formation	[105]
Mitoxantrone and pixantrone	B-cell non-Hodgkin’s lymphoma	Clinical trials	Signaling	MCU complex inhibition	[106]
GLS inhibitors	Breast and Burkitt lymphoma	Clinical trials	Biosynthesis	Reducing glutamine catabolism	[107,108]

**Table 2:** Mitochondria-targeted cancer therapy for specific transporters.

Experimental Condition	Therapeutic Agent	Treatment Method	Reference
Ovarian cancer	Paclitaxel-NPs	Chemotherapy	[45]
Ovarian cancer	Cisplatin-based peptide delivery	Chemotherapy	[46]
Several cancers	PEGylated-NPs	Drug solubility	[47]
Cancer therapy	ROS-sensitive NPs	Intracellular release	[50]
Mitochondrial disorders	Mitochondrial transcription factors	Gene therapy	[55]
Neurodegenerative diseases	Antioxidant-NPs	Oxidative stress reduction	[56]
Gene therapy	Oligonucleotide-NPs	Nucleic acid delivery	[57]
Neurodegenerative diseases	Curcumin-NPs	Antioxidant therapy	[61]
Cardioprotection	Mitochondria-targeted Peptides	Cellular uptake	[62]
Mitochondrial diseases	Mitochondrial proteins	Mitochondrial dynamics	[118]
Breast cancer	Doxorubicin-NPs	Chemotherapy	[153]
Solid tumors	Antibody-NP complexes	Targeted therapy	[154]
Cancer treatment	Mitochondria-targeted aptamers	Targeted therapy	[155]

## Data Availability

No new data were created or analyzed in this study. Data sharing is not applicable to this article.
